# The Role of PPARα in Metformin-Induced Attenuation of Mitochondrial Dysfunction in Acute Cardiac Ischemia/Reperfusion in Rats

**DOI:** 10.3390/ijms13067694

**Published:** 2012-06-21

**Authors:** Giselle Barreto-Torres, Rebecca Parodi-Rullán, Sabzali Javadov

**Affiliations:** Department of Physiology, School of Medicine, University of Puerto Rico, San Juan, PR 00936, USA; E-Mails: giselle.barreto@upr.edu (G.B.-T.); rebecca.parodi@upr.edu (R.P.-R.)

**Keywords:** heart, ischemia/reperfusion, mitochondria, metformin, AMPK, PPARα

## Abstract

Metformin, an anti-diabetic drug, exerts cardioprotection against ischemia-reperfusion (IR) through the activation of AMPK. However, the molecular mechanisms underlying these beneficial effects remain elusive. In this study, we examined the role of PPARα in mediating cardioprotective effects of metformin on mitochondria. Hearts of male Sprague-Dawley rats perfused by Langendorff were subjected to IR in the presence or absence of metformin and the PPARβ inhibitor, GW6471. IR reduced cardiac function and compromised the structural integrity of cardiac cells evidenced by increased LDH release from the hearts. In addition, IR induced mitochondrial dysfunction as evidenced by reduced respiration and increased mitochondrial permeability transition pore (PTP) opening. However, metformin-treated hearts demonstrated improved post-ischemic recovery of cardiac function and reduced cell death that were associated with increased state 3 respiration at complexes I and II (by 27% and 32%, respectively, both *p* < 0.05) and decreased PTP opening (by 27%, *p* < 0.05) compared to untreated hearts. The protective effects of metformin on cardiac function and mitochondria were blocked by GW6471. Thus, our results demonstrate that inhibition of PPARα attenuates the beneficial effects of metformin on mitochondria in acute IR.

## 1. Introduction

Cardiac ischemia/reperfusion (IR) is associated with metabolic alterations including the generation of reactive oxygen species (ROS), calcium overload, ATP depletion, low myofibrillar activity, and cell death [1,2]. Mitochondria serve as important end targets mediating cell death resulting from cardiac IR. Defects in the electron transport chain and oxidative phosphorylation resulting from cardiac IR lead to energy deprivation of the myocardium [3,4]. Perturbations in energy metabolism are associated with activation of various cell signaling pathways, one of which involves AMP-activated protein kinase (AMPK). AMPK is a serine/threonine protein kinase that acts as a master regulator of cellular energy homeostasis in the heart. Its activity can be regulated allosterically by AMP and via phosphorylation by upstream kinases such as liver kinase B1 (LKB1) and calmodulin-dependent protein kinase kinase (CaMKK) [5,6]. Stimulation of AMPK is enhanced in response to stresses such as hypoxia and IR, which deplete cellular ATP [7,8]. Activation of AMPK leads to an increase in ATP synthesis through stimulation of glucose uptake, free fatty acid oxidation (FAO), and glycolysis, and inhibition of energy-consuming anabolic pathways such as triglyceride and protein synthesis as mechanisms of ATP conservation [6,7]. Metformin, a widely used anti-diabetic drug for type II diabetes, has been shown to exert cardioprotection against IR and post-infarction heart failure associated with increased AMPK phosphorylation although the molecular mechanisms underlying its beneficial effects remain unclear [9–11].

The protective effects of AMPK on mitochondria may be mediated through several downstream targets, one of which is peroxisome proliferator-activated receptor (PPAR) gamma coactivator 1α (PGC-1α). Existing studies provide strong evidence that AMPK and a superfamily of transcriptional activators including PGC-1α and PPARα are key players of a cascade that coordinate energy metabolism in the heart [12]. PGC-1α is an integrator of the transcriptional network that regulates cardiac mitochondrial biogenesis. In addition, PGC-1α regulates genes involved in the cellular uptake of fatty acids and FAO through direct co-activation of PPARs and estrogen-related receptors [13]. PPARs represent a family of nuclear hormone receptors that serve as central regulators of cardiac fatty acid metabolism. PPAR has three isoforms (α, β/δ, and γ); PPARα is the main isoform in the heart and is a primary regulator of fatty acid metabolism. In the heart, PPARα has been previously implicated in cardioprotective signaling [14,15]. Activation of AMPK improved oxidative metabolism in adipocytes and primary muscle cells through upregulation of mitochondrial biogenesis, and increased expression of electron transport chain enzymes and upregulation of uncoupling proteins via the deacetylation [16] or direct phosphorylation of PGC-1α [17]. In addition, inhibition of hypertrophy [18] and cardiac fibrosis [19] induced by AMPK activation was associated with enhanced PPARα activity. However, the contribution of the activation of AMPK/PPARα pathway to prevent cardiac and mitochondrial dysfunctions in acute IR remains to be elucidated.

In the current study, we examined the possible role of the AMPK/PPARα/mitochondria pathway in the cardioprotective effects of metformin during acute IR in rats. Our results demonstrated that PPARα is a critical signaling molecule that mediates AMPK-induced signaling to mitochondria and improves recovery of cardiac function after IR in the heart.

## 2. Materials and Methods

Male Sprague-Dawley rats weighing 250–275 g were purchased from Charles River (Wilmington, MA, USA). All experiments were performed according to protocols approved by the University Animal Care and Use Committee and conform to the Guide for the Care and Use of Laboratory Animals published by the US National Institutes of Health (NIH Publication No. 85-23, revised 1996).

### 2.1. Langendorff Heart Perfusion and Animal Groups

**Figure 1:**
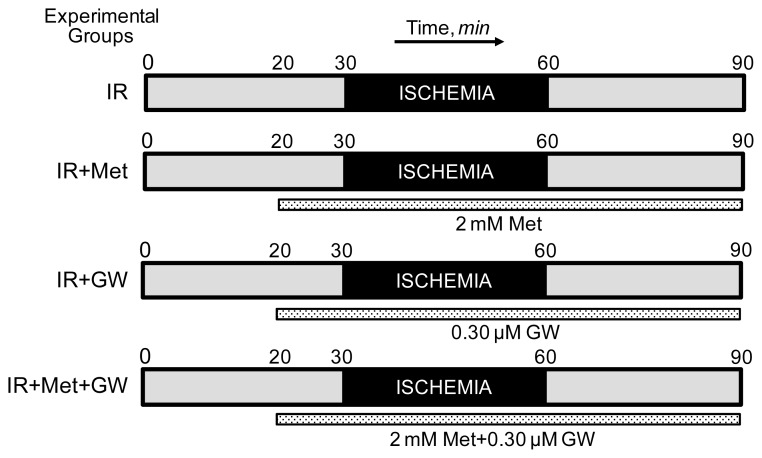
Perfusion protocols for Langendorff heart preparation. Experimental groups: (1) IR, hearts subjected to 30-min global ischemia followed by 30-min reperfusion; (2) IR+Met, hearts subjected to global ischemia followed by reperfusion in the presence of 2 mM metformin (Met); (3) IR+GW, hearts subjected to global ischemia followed by reperfusion in the presence of 0.30 μM GW6471 (GW); (4) IR+Met+GW, hearts subjected to global ischemia followed by reperfusion in the presence of 2 mM metformin and 0.30 μM GW6471.

To determine cardiac function, isolated rat hearts were perfused by the Langendorff mode as previously described [20]. Briefly, isolated hearts were perfused at a constant flow of 12 mL/min with Krebs-Henseleit buffer containing (in mM) 118 NaCl, 4.8 KCl, 1.2 KH_2_PO_4_, 1.25 CaCl_2_, 1.2 MgSO_4_, 25 NaHCO_3_, pH 7.35 ± 0.05, and 11 glucose equilibrated with 95% O_2_/5% CO_2_. A water-filled latex balloon was inserted into the left ventricle for continuous monitoring of heart rate and left ventricular developed pressure (LVDP). Initial left ventricular end-diastolic pressure (LVEDP) was set to ~5 mm Hg before the beginning of the experiment. All determinations of ventricular performance were obtained using Labscribe2 Data Acquisition Software (iWorx 308T, Dover, NH, USA). Animals were randomly assigned to one of the following treatment groups: (i) hearts subjected to global ischemia followed by reperfusion (IR group, n = 8), (ii) hearts subjected to global ischemia followed by reperfusion in the presence of the AMPK activator, metformin (2 mM, IR+Met group, n = 7), (iii) hearts subjected to global ischemia followed by reperfusion in the presence of the PPARα inhibitor, GW6471 (0.30 μM, IR+GW group, n = 6), and (iv) hearts subjected to global ischemia followed by reperfusion in the presence of 2 mM metformin and 0.30 μM GW6471 (IR+Met+GW group, n = 6). Protocols of perfusion are illustrated schematically in Figure 1. A 2 mM concentration of metformin was used in our experiments since previous studies have demonstrated that this concentration had the maximum metabolic effect without an impact on cellular energy metabolism in intact hearts [21]. Global normothermic ischemia was induced by switching off the pump and immersing the heart in buffer maintained at 37 °C after a total pre-ischemic period of 30 min. In all experiments, the ischemic period was 30 min, after which flow was restored to pre-ischemic levels and reperfusion was followed for 30 min. When metformin and/or GW6471 were present, the hearts were perfused for 10 min before ischemia and throughout the reperfusion period with the drugs dissolved in Krebs-Henseleit solution. Samples of perfusate were collected prior to ischemia and during reperfusion at indicated time points to measure the lactate dehydrogenase (LDH) activity. The hearts were used to isolate mitochondria after the corresponding protocols. LDH activity in perfusate was assessed by an enzymatic method as previously described [20].

### 2.2. Isolation of Mitochondria

To isolate mitochondria, the ventricles were cut, weighed and homogenized with a Polytron homogenizer in 5 mL of ice-cold sucrose buffer containing 300 mM sucrose, 10 mM Tris-HCl, and 2 mM EGTA; pH 7.4. The volume was made up to 40 mL with buffer containing 5 mg/mL BSA. Mitochondria were isolated from the homogenate by centrifugation at 2000× g for 2 min in a benchtop centrifuge to remove cell debris, followed by centrifugation of the supernatant at 10,000× g for 5 min to sediment the mitochondrial suspension. The pellet was then washed two times at 10,000× g for 5 min in 40 mL of BSA-free sucrose buffer. The final pellet was resuspended in 300 μL of sucrose buffer. The yield of mitochondria was 12.1 ± 0.9 mg of protein per mL. A 200-μL sample of the suspension was then used for measurement of mitochondrial respiration rates and PTP opening. A 100-μL sample was retained for assay of citrate synthase activity and protein expression.

### 2.3. Measurement of the Respiration Rates in Isolated Cardiac Mitochondria

Measurement of mitochondrial respiration was performed at 30 °C using a YSI Oxygraph (Yellow Springs, OH, USA) model 5300 equipped with a Clark-type oxygen electrode [20]. The solubility of oxygen in buffer was 230 nmol of oxygen per mL after standard calibration in water. Mitochondria were suspended in a buffer containing (in mM): 125 KCl, 20 MOPS, 10 Tris, 0.5 EGTA, and 2 KH_2_PO_4_, pH 7.2, supplemented with either of the following substrates to measure complex I- and complex II-mediated respiration rates, respectively: (i) 2.5 mM 2-oxoglutarate and 1 mM L-malate or (ii) 2.5 mM succinate and 1 μM rotenone. Respiration rates were measured in the absence (state 2) and presence (state 3) of 1 mM ADP. At the end of each run, 0.5 μM antimycin A followed by 10 mM ascorbate and 0.3 mM *N*,*N*,*N*′,*N*′-tetramethyl-*p*-phenylendiamine (TMPD) were added and the new rate of respiration measured. The respiration rates were normalized to mg of mitochondrial protein. Citrate synthase activity was determined spectrophotometrically by measuring coenzyme A formation at 412 nm [22].

### 2.4. Measurement of PTP Opening in Isolated Mitochondria

Swelling of de-energized mitochondria as an indicator of PTP opening in the presence or absence of Ca^2+^ was determined by monitoring the decrease in light scattering at 520 nm as described previously [20]. Mitochondria were incubated at 25 °C in 3 mL buffer containing 150 mM KSCN, 20 mM MOPS, 10 mM Tris and 2 mM nitrilotriacetic acid, supplemented with 0.5 μM rotenone, 0.5 μM antimycin and 2 μM A23187. Mitochondria containing ~1 mg of protein were added and swelling was initiated by progressive additions of CaCl_2_.

### 2.5. SDS-PAGE and Western Blotting

Protein concentration in homogenate and mitochondria was determined by the Bradford protein assay (Bio-Rad, Hercules, CA, USA). Equal amounts of homogenate or mitochondrial protein were resolved by SDS-PAGE, and transferred onto Amersham Hybond ECL nitrocellulose membranes (GE Healthcare Bio-Sciences Corp., Piscataway, NJ, USA). The membranes were immunoblotted with AMPK, P-AMPKα_1_^Thr172^, voltage-dependent anion channel (VDAC), LKB1, P-LKB1^Ser428^ (Cell Signalling, Boston, MA, USA), cyclophilin D (CyP-D), adenine nucleotide translocase (ANT), PPARα (Santa Cruz Biotechnology, Santa Cruz, CA, USA), PGC-1α (Abcam, Cambridge, MA, USA) or actin (Sigma-Aldrich, St. Louis, MO, USA) antibodies followed by secondary antibodies. The signals were visualized using Thermo Scientific Pierce ECL Western Blotting Detection reagents (Thermo Scientific, Rockford, IL, USA) at the VersaDoc 3000 Gel Imaging System (Bio-Rad).

### 2.6. Co-Immunoprecipitation

Protein samples were incubated with anti-PPARα antibodies (1 μg per 100 μg protein samples) overnight at 4 °C and the immunoprecipitates were harvested by protein A/G-agarose beads (Santa Cruz Biotechnology) for 3 h [23]. The immunoprecipitated complexes were washed extensively, and subjected to SDS-PAGE followed by immunoblotting for detection of the mitochondrial PTP proteins, VDAC, ANT and CyP-D.

### 2.7. Statistical Analysis

Data are presented as means ± SEM of 6–8 experiments per group. The statistical differences between groups were calculated by two-tailed Student’s *t*-test. Heart function data were compared using two-way (time and treatment factors) ANOVA and group differences were detected using a Tukey post-hoc test. Differences were considered to be statistically significant when *p* < 0.05.

## 3. Results and Discussion

### 3.1. Metformin-Induced Improvement of Post-Ischemic Cardiac Function Is Attenuated by GW6471

**Figure 2:**
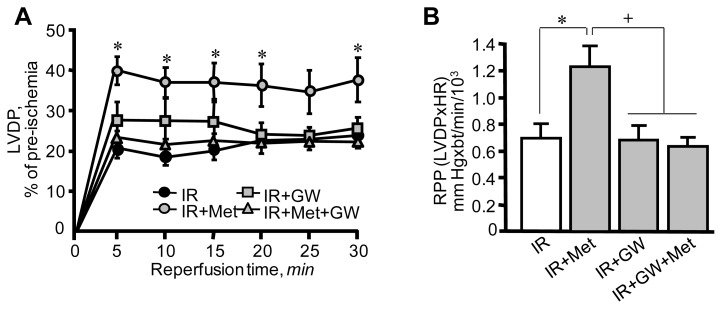
The effects of metformin (Met) on post-ischemic recovery in hearts in the presence or absence of GW6471 (GW). (**A**) Left ventricular (LV) developed pressure (LVDP) calculated as the difference between LV systolic pressure and LV end-diastolic pressure (LVEDP). Data are expressed as percent of pre-ischemic values. (**B**) Rate-pressure product (RPP), an indicator of heart performance calculated as LVDP × heart rate (HR). Data are expressed in mm Hg × beats per min. * *p* < 0.05 IR+Met *vs.* IR; ^+^ *p* < 0.05 IR+GW or IR+Met+GW *vs.* IR+Met.

**Figure 3:**
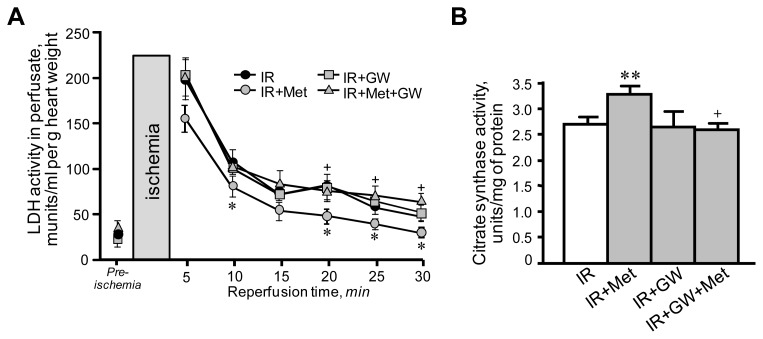
Lactate dehydrogenase (LDH) activity in eluates from hearts collected during reperfusion (**A**), and citrate synthase activity in the mitochondria (**B**) isolated from IR hearts treated with metformin (Met) in the presence or absence of GW6471 (GW). Enzyme activity is shown as munits/mL of perfusate per gram heart weight for LDH, and units per mg of mitochondrial protein for citrate synthase. * *p* < 0.05, ** *p* < 0.01 IR+Met *vs.* IR; ^+^ *p* < 0.05, ^++^ *p* < 0.01 IR+Met+GW *vs.* IR+Met.

First, we examined the cardioprotective effects of metformin on hearts subjected to IR in the presence or absence of the PPARα antagonist GW6471. IR exerted detrimental effects on cardiac performance. Hearts subjected to IR exhibited low LVDP values at reperfusion when compared to pre-ischemia (Figure 2A). In addition, the rate-pressure product (RPP), an indicator of heart performance calculated as LVDP × heart rate was lower at 30 min of reperfusion in hearts subjected to IR (Figure 2B). Notably, GW6471 *per se* caused no changes in IR-induced cardiac dysfunction. Addition of metformin to the perfusion medium (IR+Met group) significantly improved post-ischemic recovery of the IR hearts, which demonstrated great recovery of LVDP compared to IR group. The RPP was 76% (*p <* 0.05) higher in the IR+Met group compared to the IR group at 30 min of reperfusion. The beneficial effects of metformin on cardiac performance were prevented when the hearts were perfused with GW6471 and metformin simultaneously. Metformin also decreased the activity of LDH in perfusate as an indicator of necrotic cell death at reperfusion (Figure 3A). Similar to physiological parameters, metformin-induced reduction of LDH release was markedly abrogated in the presence of GW6471. The cardioprotective effects of metformin converged on mitochondria since a reduction was observed in the activity of citrate synthase, a marker of mitochondrial mass, in mitochondria isolated from IR hearts (Figure 3B). It should be noted that the citrate synthase activity may be reduced due to a direct effect of increased oxidative stress and ROS accumulation in the matrix of mitochondria at reperfusion. Treatment of the hearts with metformin in combination with GW6471 did not exert cardioprotection against IR damages.

Overall, the results of these experiments show that metformin attenuated cardiac dysfunctions associated with preserved structural integrity of cardiac cells and mitochondria during acute IR in rats. The cardioprotective action of metformin was prevented in the presence of the PPARα antagonist, GW6471.

### 3.2. Beneficial Effects of Metformin on Mitochondria Are Prevented by Inhibition of PPARα

**Figure 4:**
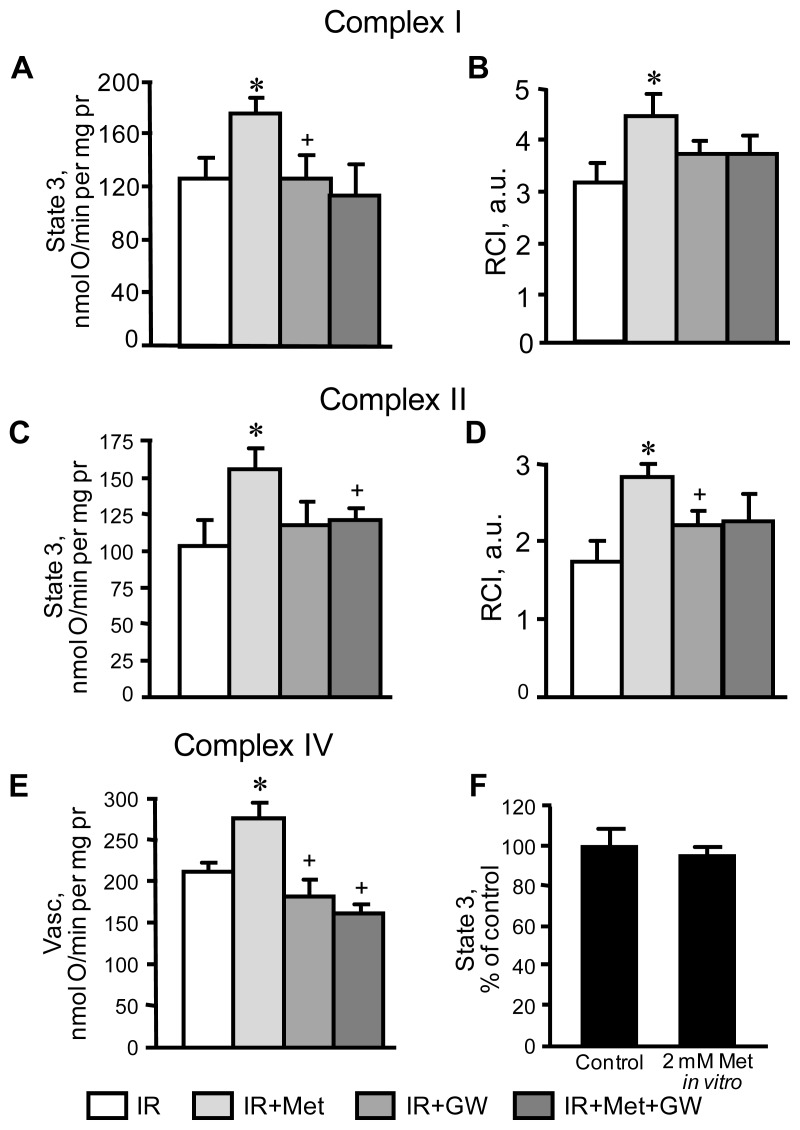
Respiration rates of mitochondria isolated from the IR hearts treated with metformin (Met) in the presence or absence of GW6471 (GW). (**A**) State 3 at complex I measured in presence of 2.5 mM 2-oxoglutarate and 1 mM L-malate as substrates; (**C**) State 3 at complex II measured in presence of 2.5 mM succinate as a substrate; (**E**) Respiration rate at complex IV measured in presence of 10 mM ascorbate and 0.3 mM TMPD; (**F**) A direct effect of metformin (2 mM) on state 3 at complex I. Panels **B** and **D** show the respiratory control index (RCI) values calculated as the ratio of state 3 to state 2. * *p* < 0.05 IR+Met *vs.* IR; ^+^ *p* < 0.05 IR+GW or IR+Met+GW *vs.* IR+Met.

In the next set of experiments, we examined whether the cardioprotective effects of metformin on mitochondria are mediated through PPARα. Mitochondria isolated from IR hearts with or without metformin and/or GW6471 treatment were used to determine respiration rates at complexes I, II and IV. Results demonstrated that basal respiration (state 2) of mitochondria at both complexes I and II was not affected in any of our experimental groups (not shown). IR markedly reduced state 3 respiration, and respiratory control index (RCI), which indicates a decrease in the efficiency of respiratory coupling and ATP synthesis. In metformin-treated hearts, state 3 respiration at complexes I and II was 31% and 51% greater (*p* < 0.05 for both), respectively, than in IR hearts (Figure 4A,C). RCI increased by 41% (*p* < 0.05) for complex I and 63% (*p* < 0.01) for complex II in metformin-treated hearts when compared to the IR group (Figure 4B,D). As shown in Figure 4E, metformin also increased state 3 by 31% at complex IV (*p* < 0.05 *vs.* IR group). The beneficial effects of metformin on mitochondrial respiration in hearts subjected to IR was prevented in the presence of GW6471 as seen by a decrease of 36% (*p* < 0.05) in state 3 compared to hearts treated solely with metformin. Previous studies demonstrated a direct inhibitory effect of metformin at a high concentration (10 mM) on complex I activity in cultured cells and isolated liver mitochondria [24,25]. To further address this concern regarding the possible direct effect of metformin on mitochondria, we examined *in vitro* the effect of 2 mM metformin on state 3 respiration rate at complex I in mitochondria isolated from the intact rat hearts. As shown in Figure 4F, the addition of metformin to mitochondrial suspensions had no impact on state 3.

Altogether, these experiments demonstrate that the beneficial effects of metformin on mitochondria respiration rates in hearts subjected to IR are mediated through PPARα.

### 3.3. Attenuation of PTP Opening Induced by Metformin Is Abrogated in the Presence of GW6471

**Figure 5:**
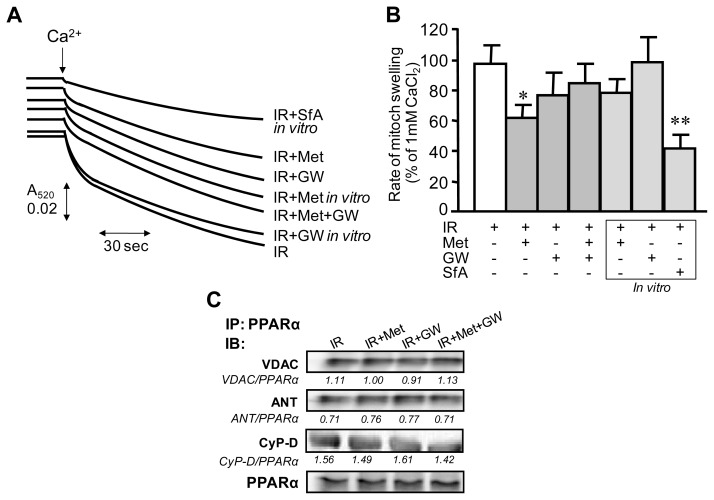
Ca^2+^-induced mitochondrial swelling as an indicator of PTP opening in the IR hearts treated *in vivo* with metformin (Met) and/or GW6471 (GW), and in mitochondria isolated from IR hearts and treated *in vitro* with Met, GW or sanglifehrin A (SfA). Measurement of the PTP under de-energized conditions was performed by monitoring the calcium-induced decrease in light scattering at 520 nm. (**A**) Original traces are shown for one mitochondrial preparation derived from hearts of IR, IR+Met, IR+GW or IR+Met+GW groups. Additionally, mitochondria isolated from hearts of the IR group were treated *in vitro* with 2 mM Met (IR+Met *in vitro*), 0.30 μM GW (IR+GW *in vitro*) or 0.5 μM SfA, an inhibitor of PTP (IR+SfA *in vitro*). To measure the effect of Met, GW, or SfA on PTP opening *in vitro*, the agents were added directly to the cuvette and swelling of mitochondria was monitored. Matrix swelling was induced by 200 μM or 1 mM (maximal swelling) Ca^2+^; (**B**) Rates of swelling of mitochondria. Data are expressed relative to the maximum rate determined at 1 mM Ca^2+^. * *p* < 0.05 IR+Met *vs.* IR; ** *p* < 0.01 IR+SfA *in vitro vs.* IR; (**C**) Representative immunoblots showing interaction between PPARα and the PTP components, VDAC, ANT and CyP-D. Heart homogenates from each group were immunoprecipitated (IP) with anti-PPARα antibodies. The complexes were subjected to SDS-PAGE followed by immunoblotting (IB) with VDAC, ANT, CyP-D or PPARα antibodies. Densitometric data for VDAC, ANT and CyP-D blots were normalized to PPARα and given below each band.

The extent of mitochondrial PTP opening was determined by measuring Ca^2+^-induced light scattering of mitochondria isolated from hearts after Langendorff perfusion. In addition, a direct effect of metformin was determined *in vitro* by addition of the drug to mitochondrial suspensions. Sanglifehrin A, a strong inhibitor of PTP opening, was used as a positive control. Results demonstrated that mitochondria isolated from the hearts subjected to IR are more prone to Ca^2+^-induced swelling (Figure 5A,B). However, metformin-treated hearts revealed 36% (*p* < 0.05) less rate of mitochondrial swelling and hence, less PTP opening than untreated IR hearts. In hearts treated with metformin in the presence of GW6471 the attenuation of PTP opening was not observed. Additionally, we evaluated the possible direct effect of metformin and GW6471 on Ca^2+^-induced swelling through *in vitro* experiments. We found that Ca^2+^-induced swelling of mitochondria is not directly affected by metformin and GW6471 (Figure 5B).

In the following groups of experiments, co-immunoprecipitation was performed to clarify whether PPARα interacts with the PTP components, VDAC, ANT and CyP-D. As shown in Figure 5C, the hearts from all 4 groups demonstrated physical association of PPARα with VDAC, ANT and CyP-D. These findings can be explained by the fact that homogenate contains an abundance of proteins and IR may possibly play a role in inducing an interaction between PPARα and PTP components. Furthermore, the interaction was not affected by metformin, GW6471, or both. Thus, these studies demonstrate that metformin-treated hearts are more resistant to PTP opening, and that the effect of metformin is abrogated by GW6471. Inhibitory effects of metformin-induced PPARα modulation on mitochondrial PTP opening are indirect.

### 3.4. Metformin Enhances AMPK Phosphorylation with No Changes in PPARα Expression

**Figure 6:**
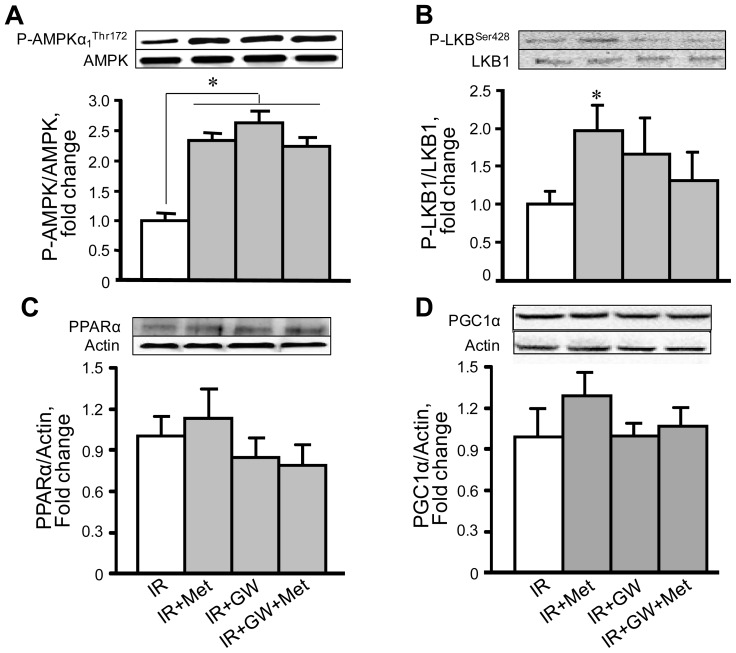
The effects of metformin (Met) on phosphorylation of AMPKα_1_^Thr172^ (**A**), LKB1 (**B**), PPARα (**C**) and PGC-1α (**D**) expression. Representative Western blot images of P-AMPKα_1_^Thr172^, AMPK, P-LKB1^Ser428^, LKB1, PPARα, PGC-1α and actin are shown for each graph (upper panels). Quantitative data are expressed as fold change relative to IR group (lower panels). * *p* < 0.05 IR+Met, IR+GW or IR+Met+GW *vs.* IR.

In the next group of experiments we investigated the effects of metformin on protein levels of AMPK and PPARα. Homogenates were isolated from the IR hearts treated with metformin and/or GW6471 and then subjected to SDS-PAGE followed by immunoblotting. Results demonstrated that metformin increased phosphorylation of AMPK at Thr172 in the hearts subjected to IR (Figure 6A). Addition of GW6471 (IR+Met+GW group) did not prevent metformin-induced phosphorylation of AMPK. In addition, metformin increased phosphorylation of LKB1, a primary upstream kinase of AMPK, although GW6471 had no effect on LKB1 activation. Metformin and GW6471 were unable to influence the expression of PPARα (Figure 6C) and PGC-1α (Figure 6D). No changes in PPARα and PGC-1α levels by either metformin, GW6471, or their combination are presumably due to shortness of the IR period. These data show that the beneficial effects of metformin on cardiac function and mitochondrial metabolism are associated with phosphorylation of AMPK which is the upstream molecule for PPARα.

Thus, the present study demonstrated that metformin improved recovery of cardiac contractility after acute ischemia in Langendorff-perfused rat hearts, and that the cardioprotective effects were associated with attenuation of mitochondrial dysfunction. Pharmacological inhibition of PPARα by GW6471 abrogated the beneficial effects of metformin on cardiac performance and mitochondrial function. Metformin is a biguanide drug that is broadly used in clinical practice to prevent hyperglycemia in patients with diabetes mellitus. In addition to its anti-hyperglycemic action, metformin also exerts cardioprotective effects and improves clinical outcomes during IR and heart failure [26]. Previous studies demonstrated that the beneficial effects of metformin are associated with its ability to activate AMPK, which significantly reduced cardiac dysfunction during *ex vivo* IR [27] and *in vivo* post-infarction heart failure [10,28,29]. Likewise, our data exhibited increased phosphorylation of AMPK at Thr172 by metformin in ischemic hearts suggesting that the activation of AMPK during acute IR is a key mechanism mediating the beneficial effects of metformin. IR compromises energy metabolism due to mitochondrial dysfunction which, in turn, affects signaling molecules that regulate energy homeostasis such as AMPK. AMPK acts a “fuel sensor” that regulates energy metabolism in the heart through the activation of transcription factors and the modulation of multiple signaling pathways [6–8] including PGC-1α [5,10,12,30]. PGC-1α is an inducible co-activator that regulates mitochondrial biogenesis through downstream transcriptional regulatory circuits, and coactivation of PPARs and estrogen-related receptors [31]. Among the three isoforms (PPARα, PPARβ/δ, and PPARγ), PPARα is the main cardiac isoform and primary regulator of fatty acid metabolism in the heart. Despite studies that have provided evidence that PGC-1α can be regulated by AMPK [17,32], there are few, if any, data on whether beneficial effects of AMPK on the ischemic heart are mediated through PPARα. Our results demonstrate that the specific potent (IC_50_ = 0.24 μM) PPARα antagonist GW6471 prevented the beneficial effects of AMPK activation on mitochondrial respiratory function and opening of the PTP. AMPK could modulate PPARα activity either directly or indirectly through PGC-1α and other signalling molecules. Activation of PPARα is most likely attributed to a post-translational modification rather than to increased protein expression of the receptor given that our model is acute IR. Indeed, PPARα contains multiple potential phosphorylation sites and it is likely that, along with AMPK, other signal transduction pathways can also modulate PPARα activity at the post-translational level. We found no differences in PPARα expression between metformin-treated and control hearts. Previous studies demonstrated that activation of PPARα by the specific agonists WY14643 and GW7647 protected hearts against IR injury and reduced infarction size in rodents [14,15]. Noteworthy, activation of PPARα by AMPK might be different from that induced by pharmacological agonists of PPARα. The next step in the AMPK/PPARα/mitochondria axis is the inhibition of IR-induced PTP opening by active PPARα. Despite studies that have shown cardioprotective effects of metformin against IR, there are few, if any, data on the effects of PPARα activation on mitochondria in acute IR. We observed inhibition of PTP formation in metformin-treated ischemic hearts that was prevented by the PPARα antagonist GW6471. PPARα may prevent PTP formation by direct interaction with the PTP components, VDAC, ANT and/or CyP-D or it can affect indirectly via activation of other signaling pathways such as PI3K/Akt and NO production [14]. Our co-immunonoprecipitation studies revealed no changes in the interactions between PPARα and VDAC, ANT or CyP-D in metformin-treated hearts (Figure 5C). These data demonstrate that the beneficial effects of the AMPK/PPARα pathway activation on mitochondrial PTP are mediated through the indirect mechanisms. Noteworthy, our *in vitro* studies revealed that metformin had no direct effects on complex I (Figure 4F) and Ca^2+^-induced swelling (Figure 5C) when the agonist was added to mitochondrial suspensions isolated from rat hearts. Metformin at a concentration of 10 mM has been previously shown to exert direct inhibitory effects on the activity of complex I, and PTP opening in cultured cancer cells [24]. Studies with isolated liver mitochondria demonstrated that a direct inhibitory effect of 10 mM metformin on complex I required incubation with mitochondria for up to 6 h indicating a very slow permeation of the drug into the mitochondria [25]. In addition, previous studies found that metformin at a concentration of 2 mM had the maximum metabolic effect without an impact on cellular energy metabolism although at high concentrations (more than 2 mM) it reduced the energy state of intact hearts [21,33]. In our studies, incubation of cultured H9c2 cardiomyoblasts with various concentrations of metformin (0.1; 0.5; 1.0; 2.0; 5.0 and 10 mM) for 1 h demonstrated that AMPK activation occurs at 2.0; 5.0 and 10 mM of the drug (*unpublished data*). Based on these studies, and due to shortness of IR, we used 2 mM metformin in our experiments. In clinical practice, metformin-HCl tablets for daily oral administration contain up to 1000 mg of the drug which is absorbed slowly within several hours and reaches a steady-state concentration in blood at 24–48 h after administration. We suggested that the heart would not accumulate (uptake) much metformin within 70 min (10 min pre-ischemia, 30 min ischemia and 30 min reperfusion) of acute IR. Noteworthy, metformin concentrations were not investigated in the heart tissue which is the limitation of the study.

Several mechanisms can be responsible for the AMPK/PPARα-mediated improvements of mitochondrial function in IR hearts. The main function of mitochondria to produce ATP from FFA in the heart is controlled, in part, at the level of expression of nuclear genes encoding enzymes that catalyze FAO. PPARα is the key transcriptional regulator of genes of FAO enzymes in the heart and its upregulation stimulates ATP synthesis [31]. Although this scenario could play an important role in a chronic model of IR or heart failure, it has less probability to take a place in acute IR when the IR period (60 min) is not sufficient for activation of gene translation and *de novo* protein synthesis. In addition, our experiments were performed using the isolated Langendorff-perfused heart model in the absence of FFA in perfusate. One of the mechanisms through which PPARα might improve mitochondrial function in IR is related to production of nitric oxide (NO). The PPARα agonist WY14643 has been shown to reduce IR injury that was associated with upregulation of NO synthase and enhanced NO production in acute IR in rats [34]. The cardioprotective effects of PPARα activation were abolished when WY14643 was administered in combination with the NO synthase inhibitor *N*-nitro-L-arginine suggesting that PPARα exerts cardioprotection via NO production. In addition to S-nitrosylation of critical proteins involved in apoptosis, NO can induce opening of mitochondrial K_ATP_ channels via the classic NO-cGMP-PKG pathway which protects against cell death by preventing mitochondrial cytochrome c release, Ca^2+^ overload, and PTP opening [35]. NO can also reduce mitochondrial ROS through direct inhibition of complex I during ischemia thereby minimizing ROS production at reperfusion [36]. Inhibition of complex I may also prevent opening of the PTP and make mitochondria more tolerant to calcium overload [37]. Notably, metformin might exert its beneficial effects on the heart in a LKB1/AMPK-independent manner. The metabolic actions of metformin in the heart muscle occurred independent of changes in AMPK activity but through p38 MAPK- and PKC-dependent mechanisms [21]. Recent studies demonstrated that metformin inhibited hepatic gluconeogenesis and reduced energy state in the liver independently of the LKB1/AMPK activation [38]. Likewise, a metformin-induced but AMPK-independent inhibition of the mammalian target of rapamycin complex 1 (mTORC1) kinase has been shown in mouse embryonic fibroblasts [39]. These studies suggest that metformin may regulate PPARα independently (or in parallel) of AMPK activation.

## 4. Conclusions

Our study demonstrates that metformin exerts cardioprotection against acute IR by improving mitochondrial function through activation of AMPK and its downstream signalling molecule, PPARα. Most likely, the effects of the AMPK/PPARα pathway activation in mitochondria are not associated with direct interactions with the PTP complex. Further studies are required for understanding the precise molecular mechanisms underlying the cardioprotective effects of the AMPK/PPARα/mitochondria pathway against acute IR damages. This study emphasizes the role of metformin-induced activation of PPARα as a potential target for the development of new therapeutic strategies aimed at the prevention of IR-induced cardiac dysfunction.
