# Expression of Concern: Barreto-Torres et al. The Role of PPARα in Metformin-Induced Attenuation of Mitochondrial Dysfunction in Acute Cardiac Ischemia/Reperfusion in Rats. *Int. J. Mol. Sci.* 2012, *13*, 7694–7709

**DOI:** 10.3390/ijms27052398

**Published:** 2026-03-05

**Authors:** IJMS Editorial Office

**Affiliations:** MDPI, St. Alban-Anlage 66, 4052 Basel, Switzerland; ijms@mdpi.com

The Editor-in-Chief of the *International Journal of Molecular Sciences* wishes to alert readers to concerns regarding apparent irregularities in the images presented in Figure 6B,C of this article [1]. An evaluation was conducted by the Editorial Board in consultation with the authors; however, as the original image data are no longer available for verification, the concerns could not be conclusively resolved. This notice is issued to ensure transparency and to maintain the integrity of the scholarly record. No further editorial action is planned at this time. The journal will reconsider the matter should additional information become available.
